# Multiomics Reveals the Key Microorganisms and Metabolites in the Resistance to Root Rot Disease of *Paris polyphylla*

**DOI:** 10.3390/genes15010021

**Published:** 2023-12-22

**Authors:** Ting Ye, Hailan Su, Guohua Zheng, Hongyan Meng, Wenhua Wang, Ying Guo

**Affiliations:** 1 Fujian Key Laboratory of Subtropical Plant Physiology and Biochemistry, Fujian Institute of Subtropical Botany, Xiamen 361006, China; yt881115@126.com (T.Y.); mhy1984@126.com (H.M.); wangwenhua0629@163.com (W.W.); 2 Institute of Crop Sciences, Fujian Academy of Agricultural Sciences, Fujian Germplasm Resources Center, Fuzhou 350000, China; suhailan2019@163.com; 3 Plant Introduction & Quarantine Base and Plant Product Key Laboratory of Xiamen City, Xiamen Overseas Chinese Subtropical Plant Introduction Garden, Xiamen 361002, China; zhengguohua413@aliyun.com

**Keywords:** *Paris polyphylla*, microbiome, transcriptome, metabolome, root rot disease

## Abstract

Root rot of *Paris polyphylla* has received widespread attention due to its threat to yield and leads to serious economic losses. However, the relationship among the rhizosphere microbial community, metabolites and root rot disease remained largely unexplored. Herein, we used integrated 16S rRNA, ITS, RNA sequencing and UPLC-MS/MS to systematically investigate the differences between healthy and diseased *P. polyphylla*. We found that root rot reduced the microbial diversity in the diseased *P. polyphylla* compared with the healthy control. The relative abundance of the bacterial phylum *Actinobacteria* increased in the diseased rhizome of *P. polyphylla*. For the fungal community, root rot disease contributed to an increased relative abundance of *Ascomycota* and decreased *Glomeromycota* at the phylum level. The transcriptomic results showed that the differently expressed genes were significantly enriched in the “Biosynthesis of various alkaloids”, “flavonoid biosynthesis” and “isoflavonoid biosynthesis” and “Phenylpropanoid biosynthesis” was dramatically enriched in healthy *P. polyphylla* compared with that in diseased *P. polyphylla.* Likewise, the metabolomic results showed that the biosynthesis of secondary metabolites and metabolic pathways was found to be significantly enriched by differential metabolites. Taken together, the study of combining metabolomics with microbiomes can help us enhance our understanding of the mechanisms of plant resistance to root rot disease, thereby discovering specific metabolites and microorganisms that can resist pathogen infection in *P. polyphylla*.

## 1. Introduction

*Paris polyphylla* is a perennial herbaceous plant belonging to the *Liliaceae* family and Paris genus [1]. In China, although there are many types of *P. polyphylla*, only two are recorded in the Pharmacopoeia. One is *polyphylla Smith var. yunnanensis*, also called “Dian chonglou” in Chinese, and the other is *Paris polyphylla* Smith *var. chinensis*, also called “Hua chonglou” [2,3]. The dry rhizomes of *P. polyphylla* are used in Chinese herbal medicine [4]. This medicine is the main ingredient of the hemostatic prescription “Yunnan Baiyao”. It has important clinical effects in treating snake bites, fractures, mumps, tumors, analgesia and other aspects [5]. In recent years, pharmacological studies have shown that *P. polyphylla* has various pharmacological activities, including anticancer, anti-inflammatory, antibacterial and immunomodulatory activities [6].

However, with the rapid development of the planting industry and changes in ecological and environmental factors such as blind introduction and the planting mode, the quality of *P. polyphylla* has deteriorated, and the disease of *P. polyphylla* has become increasingly prominent, especially root rot [7]. During the long growth process of *P. polyphylla*, any diseases can lead to crop failure, which is the highest risk source for *P. polyphylla* cultivation and seriously restricts the development of related industries. The plant microbiota has a significant impact on the health of plants, both at the root (underground) and the above-ground biomass [8]. The microbial community on the root surface and inner circle is influenced by the plant roots. Soil microbial communities are considered essential biological processes for maintaining soil health and suppressing plant diseases [9]. The root microbiota plays an important role in many aspects of plant growth and health, including providing nutrients for plants, stimulating seed germination, promoting abiotic stress resistance, triggering plant system defense and improving antibiotic function against pathogens [10]. More and more studies have shown that the root microbiota can resist stress [11]. Recent studies have shown that rhizosphere microbiota can resist severe disease outbreaks, such as soil-borne diseases caused by *Fusarium* and *Rhizoctonia* [12]. Overall, it is widely believed that there is a close connection between plant root microbiota and plant disease progression [13]. However, there are few reports on the root microbiota after the outbreak of root rot, and this has never been reported in *P. polyphylla*. The transcriptome can effectively explore the structure and expression of genes; meanwhile, the metabolomics can investigate the differentially abundant metabolites that affect the metabolic status of an organism or cell at a specific time [14]. In recent years, conjoint analysis based on multi-functional “omics” data has proved to be a powerful tool for clarifying different aspects of plant developmental biology and environmental responses. The network analysis of metabolomics and transcriptomics has become a powerful tool for studying new genes and pathways in plants; thus, it also helps to explore new pathways for the formation of root rot *P. polyphylla*.

We assume that bacterial community structures and specific metabolites are related to the plant health status. Identifying beneficial bacteria that distinguish healthy and diseased *P. polyphylla* would be the first step to developing and utilizing local soil microbiota to protect *P. polyphylla* against disease. In the current study, healthy and diseased *P. polyphylla* were selected as experimental materials in order to have a better understanding of the differential microbial, metabolic and transcriptomic profiles of the rhizome and to clarify the unique microorganism, metabolites and genes affected by root rot disease. This study reveals the metabolic changes and transcriptional regulation of healthy and root rot diseased *P. polyphylla* through comprehensive metabolic analysis, providing a basis for our understanding of the pathogenesis of *P. polyphylla*.

## 2. Materials and Methods

### 2.1. Plant Materials and Treatments

The plants of *P. polyphylla* were grown under natural conditions in Shizhong Town, Longyan City, Fujian province, under the Chinese herbal medicine planting base (16°23′25″~116°24′41″ E, 39°55′19″~39°55′56″ N). The rhizomes of the 5-year-old *P. polyphylla* were collected on July 20, 2021. A five-point sampling method was adopted to select the rhizosphere of 5-year-old *P. polyphylla* in the planting base; then, the six healthy samples and six disease samples were collected, respectively. A total of 1 cm^3^ of the rhizosphere was cut with disinfection blades after disinfection (disease samples were selected at the junction of disease and health). There were six replicates in each group. At the same time, the soil within a 5 cm diameter around the rhizosphere was collected to determine the soil microbial diversity. The roots were shaken to remove loose soil, and the remaining adhering soil was carefully collected with a sterile brush to represent the rhizosphere soil. The soil of the same base where *P. polyphylla* was not planted was used as a blank control.

### 2.2. Isolation and Identification of the Pathogen

After washing the roots of the diseased plant with distilled water, they were naturally dried. The blade was disinfected with 75% ethanol and the samples were cut into 5 mm thin slices. The sample was soaked in 2% sodium hypochlorite for 3 min, followed by 75% ethanol for 30 s, and rinsed repeatedly with sterile water three to four times. Then, the diseased samples were placed on a PDA medium including 0.1% streptomycin and cultured at 28 °C for 3–5 days. Next, after the diameter of the colony reached 2–3 cm, a small amount of mycelium was taken from the edge of the colony and placed in the center of the PDA culture medium. The pure colony was obtained after incubation at 28 °C for 48 to 72 h, and the strain was transferred to a PDA tube. The mycelium was stored in a refrigerator at 4 °C.

The activated culture was sub-cultured on a PDA medium at 28 °C, and the mycelium was cultured on a PDA medium for 5 days. The mycelium DNA was extracted with the Ezup column fungal genomic DNA extraction kit. The PCR amplification was carried out with ITS1/ITS4 primers (ITS1: TCCGTAGGGTGAACCTGGG; ITS4: TCCTCCGCTTATTGATATATAGC). Finally, the BLAST identification was performed on the sequencing results.

### 2.3. DNA Extraction and Sequencing

The genomic DNA of the soil and rhizome sample was extracted by the CTAB method, and the purity and concentration of DNA were detected by agarose gel electrophoresis. Then, the DNA was taken in a centrifuge tube and diluted to 1 ng/μL with sterile water. The bacterial diversity identification region is the 16S V3-V4 region (primers: 515F: GTGYCAGCMGCCGCGGTAA; 806R: GTGYCAGCMGCCGCGGTA) [15,16]. ITS genes of distinct regions were amplified using the specific primer ITS5-1737F and ITS2-2043R. After full mixing, the PCR products were detected by 2% agarose gel electrophoresis. For the target strip, the gel recovery kit provided by Qiagen Company was used to recover the products. The TruSeq ^®^ DNA PCR-Free Sample Preparation Kit was used for library construction. The constructed library was quantified by Qubit and Q-PCR, and after passing the library, NovaSeq6000 was used for machine sequencing.

### 2.4. Genome Sequencing

The raw data were preprocessed to remove low-quality reads using Qiime (V1.9.1). We utilized the Uparse algorithm Cluster all Effective Tags of all samples and, by default, clustered the sequence into OTUs (Operational Taxonomic Units) with 97% consistency (Identity). The Mothur method and SILVA138.1 were used to perform the species annotation of OTUs sequences. The SSUrRNA database was used for species annotation analysis to obtain taxonomic information. Finally, the data of each sample were homogenized, with the minimum amount of data in the sample as the standard. Subsequent α diversity and β diversity analyses were based on the homogenized data.

The fastp was used to filter the raw data, mainly to remove reads with adapters; when the N content in any sequencing reads exceeds 10% of the base number of the reads, the paired reads are removed. When the number of low-quality (Q ≤ 20) bases contained in reads exceeds 50% of the read bases, this paired read will be removed.

### 2.5. Metabolome Measurement

A widely targeted metabolomics method was used to determine the metabolites in the healthy and disease groups. Metabolite extraction, detection, identification and quantification were carried out at Wuhan MetWare Biotechnology Co., Ltd. (Wuhan, China) based on the methods described by Chen et al. (2013) [17]. Briefly, 100 mg of the tissue sample ground with liquid nitrogen was extracted with 70% aqueous methanol. The sample extracts were analyzed using a liquid chromatography–electrospray ionization–mass spectrometry (LC-ESI-MS/MS) system.

The operating parameters of the ESI source were a source temperature of 500 °C, an ion spray voltage (IS) of 5500 V (positive ion mode)/−4500 V (negative ion mode), ion source gas I (GSI), gas II (GSII) and curtain gas (CUR) set to 50, 60 and 25 psi, respectively, and a high collision activation dissociation (CAD). QQQ scanning was an MRM experiment conducted with collision gas (nitrogen) set to medium. DP (clustering potential) and CE (collision energy) were optimized for a single MRM transition. A specific set of MRM transformations were monitored for each period based on the metabolites eluted during this period.

### 2.6. Transcriptome Analysis

The total amount of RNA in each sample is 1 µg, which serves as the input material for RNA sample preparation. The sequencing library using the NEBNext ^®^ UltraTM rnlibrary Prep Kit for Illumina^®^ (NEB, Lake Forest, IL, USA) was generated according to the manufacturer’s recommendations. The cDNA library was sequenced on the Illumina sequencing platform of Metware Biotechnology Co., Ltd. (Wuhan, China). 

### 2.7. Statistical Analysis

The Uparse algorithm (Uparse v7.0.1001, http://www.drive5.com/uparse/, accessed on 18 August 2013) was used for all samples of all the effective clustering tags, and species annotation was performed on OTUs sequences using the Mothur method and the SSUrRNA database of SILVA138.1 (http://www.arb-silva.de/, accessed on 27 August 2020). The PCA diagram was drawn using R software (Version 4.1.2). For two-group analysis, differential metabolites were determined by VIP (VIP ≥ 1) and absolute Log2FC (|Log2FC| ≥ 1.0). The significance for pathways with significantly regulated metabolites was determined by the hypergeometric test’s *p*-values. The difference analysis of α and β diversity was analyzed with a parametric test and non-parametric test, respectively, and a T-test and Wilcox test were used. For the samples with biological replicates, DESeq2 was used to analyze the differential expression between the two groups.

## 3. Results

### 3.1. Morphology of Healthy and Diseased P. polyphylla and Isolation of Pathogens

**Figure 1:**
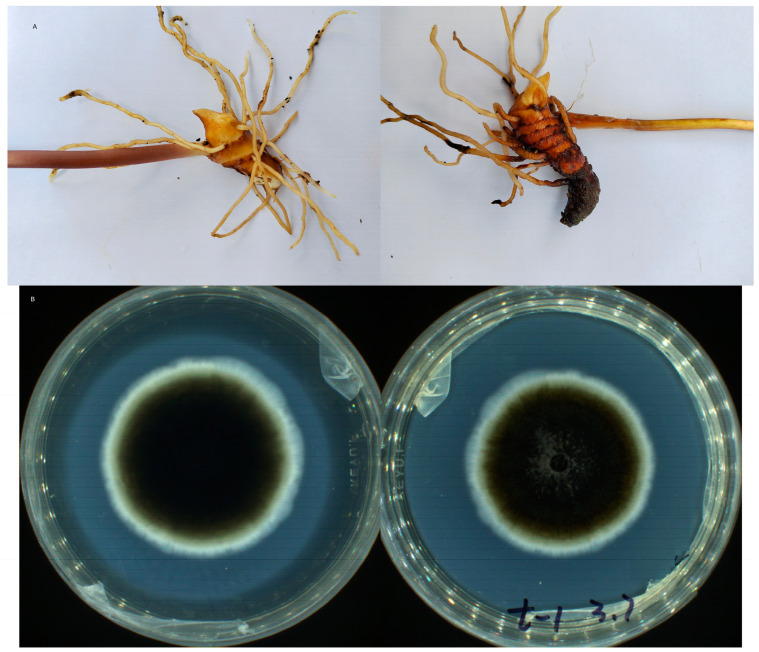
Phenotypes of rootrot diseased and healthy *P. polyphylla* and isolation of pathogenic bacteria. (**A**) Overall phenotype of root rot and healthy *P. polyphylla* plants. (**B**) Isolation of *Plectosphaerella.* t-1 was represented the number of subcultured bacterial, 3.7 was represented the date.

For a more visual view of the situation of root rot in *P. polyphylla*, the morphology of healthy and diseased rhizomes is shown in Figure 1A. There was a significant difference in appearance between healthy and diseased *P. polyphylla* in the field: chlorosis was obvious on diseased plants, while the healthy plants grew well. The healthy plants exhibited normal growth with no symptoms of necrosis on the root; however, the diseased parts of the rhizomes were obviously black and rotten, the lateral roots were black and the basic phloem of the stems was damaged. Furthermore, the pathogen was identified as *Plectosphaerella cucumerina* based on the morphology (Figure 1B). The *Plectosphaerella* genus colonies isolated on PDA were flat, slimy, appressed and grey black, with a sparse aerial mycelium and short hairy hyphae.

### 3.2. Differences in Microbial α- and β-Diversity of Healthy and Diseased P. polyphylla

**Figure 2:**
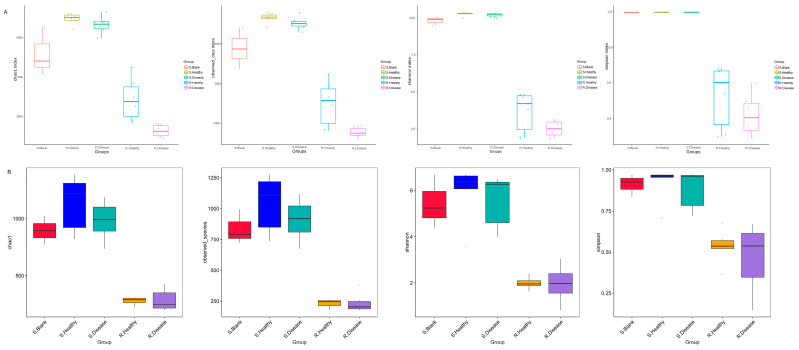
α-diversity indices of the bacteria (**A**) and fugal (**B**) communities of root rot diseased and healthy *P. polyphylla*. α-diversity indices are composite indices reflecting abundance and consistency measured on the basis of Observed species, Shannon, Chao1, and Simpson indices.

Bacterial communities associated with the rhizosphere and bulk soil of healthy and diseased *P. polyphylla* were characterized based on the V3–V4 region. Next, we assessed the species diversity, represented by the Chao1, Shannon, and Simpson indices. As shown in Figure 2, compared with the healthy control, the diseased bacterial Chao1 (*p* = 0.005), ACE (*p* = 0.009) and observed OTUs (*p* = 0.009) index were significantly decreased in diseased rhizome of *P. polyphylla* compared with the healthy control.

**Figure 3:**
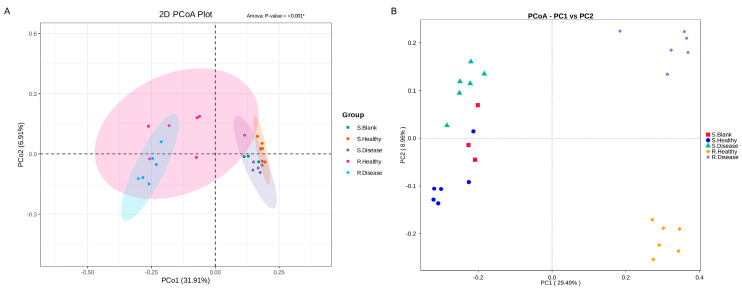
The principal co-ordinates analysis (PCoA) pairwise comparisons of microbial communities in root rot diseased and healthy *P. polyphylla*. (**A**) Bacterial PCoA; (**B**) fungal PCoA. S. Blank: blank control for soil samples; S. Healthy: healthy control for soil samples; S. Disease: diseased soil samples; R. Healthy: healthy control for rhizome samples; R. Disease: diseased rhizome samples.

To further measure the microbial differences on healthy and diseased *P. polyphylla*, principal co-ordinates analysis (PCoA) at the OUT level based on the Bray–Curtis distance was performed. In Figure 3, the PCoA analysis revealed that the soil root zone and rhizomatic bacterial communities formed four different groups. The PCo1 and PCo2 explained 31.91% and 6.91%. In addition, the PCoA analysis indicated that all samples were divided into four groups of the fungal community. PCo1 and PCo2 explained 29.49% and 8.96% of the total variation in the fungal community, respectively. These results revealed that the rhizomatic bacterial communities were significantly changed by the disease.

### 3.3. Differences in the Microbial Community of Healthy and Diseased P. polyphylla

**Figure 4:**
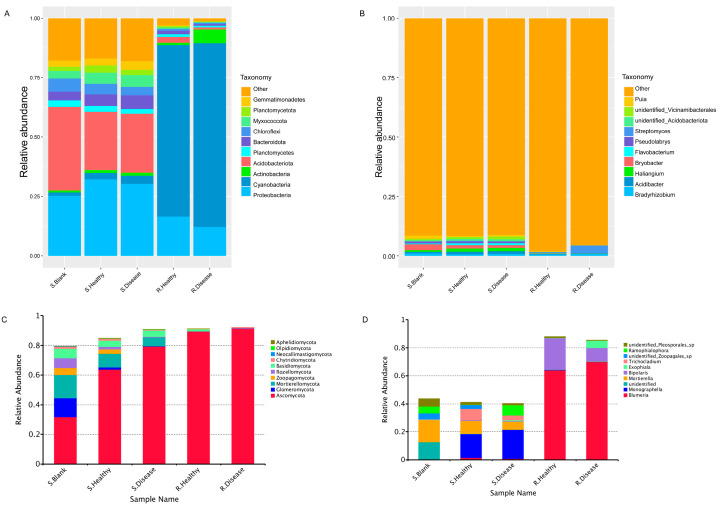
Relative abundance (%) plots of the microbial communities of root rot diseased and healthy *P. polyphylla.* The top 10 most abundant bacteria at the phylum level (**A**) and genus level (**B**) are represented. The top 10 most abundant fungi at the phylum level (**C**) and genus level (**D**) are represented.

The relative abundance (RA) of the top 10 bacterial (Figure 4A,B) and fungal (Figure 4C,D) phyla and genera was observed across all the samples. At the phylum level, Proteobacteria, Cyanobacteria and Acidobacteriota were most dominant in both heathy and diseased groups. Both R. Healthy and R. Disease samples were represented mainly by the phyla of Cyanobacteria (72.17% and 77.35%), Proteobacteria (16.52% and 12.15%) and Acidobacteriota (0.99% and 5.76%). In contrast, S. Healthy and S. Disease samples were dominated by Proteobacteria (32.23% and 30.34%), Acidobacteriota (24.48% and 24.75%) and Bacteroidota (3.59% and 4.82%). The results showed that the root rot disease of *P. polyphylla* resulted in significant changes in the microbial community composition of the root zone soil and rhizome at the phylum and genus levels compared to the healthy control. In other words, compared with the healthy control, root rot disease significantly decreased the RA of *Gemmatimonadetes* (23.37%) in the S. Disease vs. S. Healthy group. The RA of diseased *Actinobacteria* increased in the rhizome of *P. polyphylla*. For the fungal community, all the diseased and healthy samples were dominated by *Ascomycota*, *Glomeromycota*, *Mortierellomycota*, *Rozellomycota* and *Basidiomycota.* Likewise, root rot disease contributed to the increased RA of Ascomycota and decreased Glomeromycota at the phylum level.

**Figure 5:**
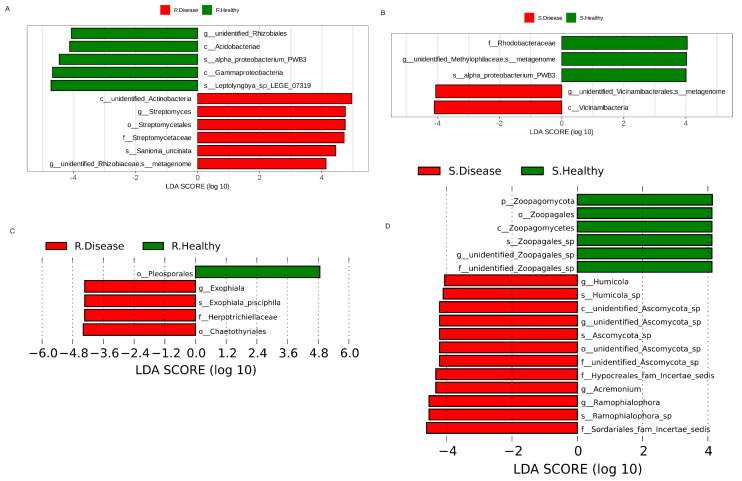
The LDA values of bacterial (**A**,**B**) and fugal (**C**,**D**) communities with significant abundance differences in root rot diseased and healthy *P. polyphylla*.

The linear discriminant analysis effect size (LEfSe) was used to find the bacterial biomarkers with significant differences between the healthy and diseased *P. polyphylla* (Figure 5). There were 11 bacterial biomarkers found in R. Healthy vs. R. Disease, including Acidobacteriae, Gammaproteobacteria, unidentified_Rhizobiales, Streptomyces and Proteobacterium. Five bacterial biomarkers were found in S. Healthy vs. S. Disease, including *Proteobacterium*, *Rhodobacteraceae* and *Vicinamibacteria* (Figure 5A). The results showed that five fungal biomarkers were found in R. Healthy vs. R. Disease, including *Ascomycota*, *Gammaproteobacteria*, *Streptomyces* and *Proteobacterium.* Eleven fungal biomarkers were found in S. Healthy vs. S. Disease, including *Zoopagomycota*, *Zoopagales*, *Zoopagomycetes*, *Humicola*, *Ascomycota* and *Acremonium.* Furthermore, the top 10 differentially abundant bacterial genera and species are shown in Table 1. The genera include unidentified_*Rhizobiales*, *Lysinibacillus*, *Anaeromyxobacter* and *Halomonas* and the species include unidentified_*Rhodospirillaceae*, *Pirellula*, *unidentified_Chloroflexi*, *unidentified_Alphaproteobacteria*, *Ferruginibacter* and *Luteitalea.*

### 3.4. Metabolomic Changes between the Healthy and Diseased P. polyphylla

**Figure 6:**
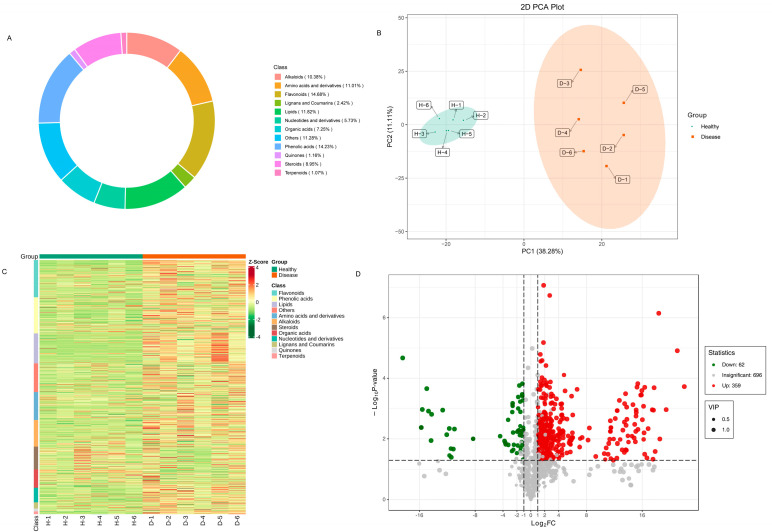
Differentially accumulated metabolites in healthy and diseased *P. polyphylla.* (**A**) Circular graph for metabolite classes; (**B**) principal component analysis (PCA) analysis pairwise comparisons of differential metabolites; (**C**) heatmap of accumulated metabolites; (**D**) volcano plots for differentially accumulated metabolites.

In order to comprehensively understand the metabolites present in healthy and diseased samples, a systematic metabolic profiling analysis was conducted. The ion flow diagrams of all samples and the multimodal diagrams of MRM metabolite detection are shown in Supplementary Figure S1. The metabolites cover eight classes, including 14.68% flavonoids, 14.23% phenolic acids, 11.82% lipids, 19.25% terpenoids, 11.01% amino acids and derivatives, 10.38% alkaloids, 8.95% steroids, 7.25% organic acids, 5.73% nucleotides and derivatives, 2.42% lignans and coumarins, 1.16% quinones, 1.07% terpenoids and 11.28% others (Figure 6A).

Principal component analysis (PCA) analysis was utilized on the metabolites to reveal the metabolite profiles of healthy and diseased *P. polyphylla*. As shown in Figure 6B, it has been shown that metabolites from different parts of healthy and diseased *P. polyphylla* were clearly separated in the score plots, where the first principal component (PC1) was plotted against the second principal component (PC2). PC1 and PC2 represented 38.28% and 11.11% of the total variation, respectively. These results suggest significant biochemical differences between healthy and diseased *P. polyphylla*. The accumulation pattern of metabolites among samples could be visualized through a heatmap hierarchical cluster analysis (Figure 6C). Furthermore, Volcano plots were utilized to depict the differently expressed metabolites between healthy and diseased *P. polyphylla*. There were 421 differential metabolites detected in the healthy vs. disease group, in which there were 359 upregulated and 69 downregulated metabolites (Figure 6D).

### 3.5. Transcriptomic Profiling of Healthy and Diseased P. polyphylla

**Figure 7:**
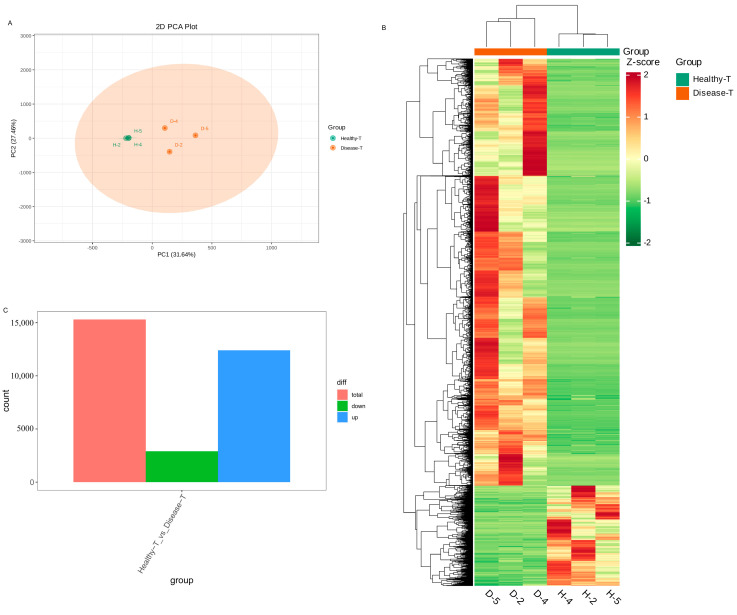
Differentially expressed genes (DEGs) in healthy and diseased *P. polyphylla.* (**A**) PCA analysis pairwise comparisons of DEGs; (**B**) heatmap of DEGs; (**C**) barplot of DEGs.

To clarify the differentially expressed genes (DEGs) of the transcriptomic differences detected in the healthy and diseased *P. polyphylla*, transcriptome sequencings of the rhizome were carried out. In Figure 7A, PC1 and PC2 represented 31.64% and 27.46% of the total variation, respectively. Furthermore, the accumulation pattern of DEGs between healthy and diseased *P. polyphylla* samples could be visualized through a heatmap hierarchical cluster analysis (Figure 7B). There were 15,305 DEGs detected in the healthy vs. disease group, in which there were 12,401 upregulated and 2904 downregulated DEGs (Figure 7C). The top 20 DEGs are shown in Table 2.

### 3.6. Differentially Accumulated Metabolites in Healthy and Diseased P. polyphylla

**Figure 8:**
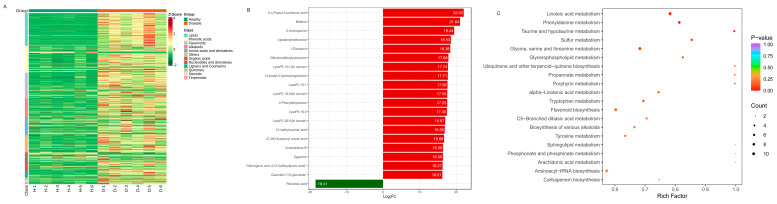
The most significantly differentially accumulated metabolites in healthy and diseased *P. polyphylla.* (**A**) Heatmap of differentially accumulated metabolites; (**B**) top 20 differentially accumulated metabolites; (**C**) KEGG pathway enrichment (**A**–**C**) of differentially accumulated metabolites.

The differential levels of secondary metabolites in different groups of healthy and diseased *P. polyphylla* are shown in Figure 8A. As the color changed, there were significant differences in the levels and types of metabolites between different groups. The 20 compounds that were the most significantly different (VIP > 1 and top 20) in all comparisons were obtained (Figure 8B). The top 20 DAMs are shown in Table 3. The major differential categories were Alkaloids, Lipids, Phenolic acid, Terpenoids, Nucleotides and derivatives, Amino acids and derivatives and flavonoids. The category of lipids includes upregulated LysoPC 22:5 (2n isomer), LysoPC 15:1, 13-methylmyristic acid, LysoPA 16:0, LysoPC 19:2 (2n isomer), LysoPE 15:1 (2n isomer), 1-Eicosanol and downregulated Ricinoleic acid. The category of alkaloids includes upregulated N-Acetyl-5-hydroxytryptamine, Dihydrocaffeoylputrescine, Betaine, Veramadines B, Zygacine and 2-Phenylethylamine.

### 3.7. Metabolic Pathway Analysis and Changing Trends of DAMs and DEGs in P. polyphylla

**Figure 9:**
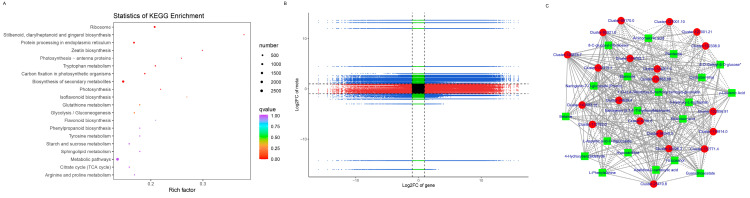
KEGG pathway enrichment of DEGs and nine-quadrant plot. (**A**) KEGG analysis of DEGs; (**B**) nine-quadrant plot. (**C**) Network diagram between DEGs and DAMs. Metabolites are shown in green squares and genes are shown in red circles. The solid line represents the positive correlation and the dashed line represents the negative correlation.

To understand the metabolism relations among the differential metabolites and genes between healthy and diseased *P. polyphylla*, we further analyzed the functional involvement of the differential metabolites and genes in different pathways by mapping them to the KEGG database. In Figure 8C, the results showed that the biosynthesis of secondary metabolites and metabolic pathways was found to be significantly enriched by differential metabolites. Several pathways are enriched by DEGs, including Flavonoid biosynthesis, Phenylalanine metabolism, Aminoacyl−tRNA biosynthesis and Glycine, serine and threonine metabolism (Figure 9A).

### 3.8. Integrative Analysis of DEGs and DAMs Reveals the Differential Regulatory Network of Metabolites Biosynthesis in P. polyphylla

All the DEGs and DAMs were subjected to correlation analysis. The difference multiples between DEGs and DAMs were shown in a nine-quadrant plot (Figure 9B), which was divided into one to nine quadrants from left to right and from top to bottom. The changes in metabolites may be positively regulated by genes, which have the same differential expression pattern and positive correlation in quadrants 3 and 7. There was a negative correlation between the DEGs and DAMs in quadrants 1, 2 and 4, and the expression abundance of metabolites was higher than that of genes. The expression abundance of metabolites in quadrants 6, 8 and 9 was lower than that of genes, and there were negatively correlated genes and metabolites. There were 3909 genes corresponding to 93 metabolites in the 13, 7 and 9 quadrants of healthy and diseased *P. polyphylla*. Furthermore, we recruited the top 20 DEGs and DAMs to perform the network analysis, which is shown in Figure 9C.

## 4. Discussion

Interestingly, plant metabolites can shape the plant microbiome, which can also affect the host’s metabolome [18,19,20,21]. The study of the association between metabolomics and microbiomes will provide a deeper understanding of the important relationship between specific metabolites and microbiomes. Due to the long growth cycle of *P. polyphylla*, it is prone to infection by various pathogenic bacteria. The root rot disease is the most serious disease of *P. polyphylla*, mainly infecting the roots of these plants, leading to root rot and irreversible wilting, ultimately leading to withering and death. The soil microbial community is influenced by various factors such as the plant type, climate, soil properties and agricultural practices [22]. In this study, multiomics analyses were used to find out the potential molecular processes and beneficial microbiota in the soil and rhizome of *P. polyphylla*. Therefore, our research indicated that root rot not only alters the transcription and functional metabolite content of the rhizome but also affects the microbial community diversity, assembly and function of the rhizome.

As is well known, the root microbiota are related to plant health, and the invasion of pathogens in plants can promote the enrichment of a group of beneficial root microbiota [19]. In our study, in the comparison between the healthy control and the diseased bacterial Chao1, it was observed that the OTUs index was significantly decreased in the rhizome of diseased *P. polyphylla*. Highly diverse microbial communities are often more complex, with greater functional redundancy and inter-boundary correlations [23]. As previously described, a decrease in microbial diversity was found in diseased banana plants [24], while a higher microbial diversity was found in healthy beets [25], beans [14] and chili peppers [26]. In addition, the reduction in diversity promotes the invasion of potential pathogenic bacteria and fungi into soil- or plant-related microbial communities [27]. The diversity index of pathogenic bacteria significantly decreased, indicating a low diversity of diseased plants.

*Actinobacteria* are a type of microorganism with biological control effects, and researchers have conducted extensive research on *Actinobacteria* [27]. *Actinobacteria* can produce a large variety of bioactive compounds such as antifungal and antibacterial compounds, iron carriers or plant growth regulators, which have been developed for agricultural use and inhibit the growth of pathogens in soil. It has been proven that certain types of *Actinobacteria* are important in the rhizosphere, where they protect roots from invasion by pathogenic fungi and may promote plant growth [28,29]. Some *Actinobacteria* are also believed to promote plant growth by forming symbiotic relationships with crop plants and colonizing their internal tissues without causing disease symptoms [30]. In this study, the RA of *Actinobacteria* being increased in the diseased rhizome of *P. polyphylla* indicated their sensitivity to root rot disease, and the plant may recruit the *Actinobacteria* to resist root rot disease.

In the current study, healthy and root rot samples were isolated from Bray Curtis and UniFrac PCoAs, indicating that these samples contain different microbial groups. To further determine that the microbiome plays an important role in this process, we compared the microbial community composition of healthy and root rot samples. Both R. Healthy and R. Disease samples were represented mainly by the phyla of *Cyanobacteria* (72.17% and 77.35%), *Proteobacteria* (16.52% and 12.15%) and *Acidobacteriota* (0.99% and 5.76%), which showed that the dominant soil phyla were not changed by root rot, although the proportions of these dominant phyla were changed. *Cyanobacteria* improve soil fertility and crop productivity by fixing atmospheric nitrogen, dissolving phosphate and releasing nutrients [31].

The resistance of plants to abiotic and biotic stresses was determined by the genes enriched in the “Phenylpropanoid biosynthesis” and biosynthesis of secondary metabolites [32]. This study revealed significant differences in the key enriched genes in plant–pathogen interactions, phenylpropanoid biosynthesis and secondary metabolites pathways between healthy and diseased roots of *P. polyphylla* through RNA Seq map analysis. A previous study has shown that the biosynthesis of plant hormones can improve the plant resistance to disease by activating the phenylpropanoid pathway [33]. Therefore, the biological pathways involved in hormone signal transduction are related to plant disease resistance [34]. In the present study, the number of DAMs in the flavonoid category was the highest, with 57 downregulated and 5 upregulated DAMs in diseased *P. polyphylla* compared with healthy *P. polyphylla*. Flavonoids are the most explored secondary metabolites in the plant defense system. Plants can activate the entire or partial network for defense, and different activation modes can be observed when plants encounter biological or environmental stress [35]. It can be indicated that the flavonoid metabolism may positively dominate the resistance of *P. polyphylla* against root rot disease.

Although metabolites associated with the root rot diseased *P. polyphylla* were identified in this study, the sample size was limited to provide a more comprehensive understanding of the metabolic diversity present in the disease. More effort can be put into mining the metabolic diversity of root rot diseased *P. polyphylla*, and the specific molecular regulation mechanism needs to be explored in root rot diseased *P. polyphylla*.

## 5. Conclusions

The current results provide solid evidence showing the difference in the microbiology community and metabolites that was caused by the root rot disease in *P. polyphylla*. The results of 16S rRNA and ITS sequencing revealed that the rhizomatic bacterial communities were significantly changed by the disease. The diseased bacterial Chao1 (*p* = 0.005), ACE (*p* = 0.009) and observed OTUs (*p* = 0.009) indexes were significantly decreased in the diseased rhizome of *P. polyphylla*. It has been found that *Cyanobacteria* increased by 5.18%, *Proteobacteria* decreased by 4.37% and *Acidobacteriota* increased by 4.77% in the diseased rhizome of *P. polyphylla* compared with that of healthy *P. polyphylla.* The metabolome data indicated that root rot disease significantly influences the alkaloidal and flavonoid metabolism of the samples between healthy and diseased *P. polyphylla*. The study of combining metabolomics with microbiomes enhances our understanding of the mechanisms of plant resistance to root rot disease, thereby discovering specific metabolites and microorganisms that can resist pathogen infection in *P. polyphylla*.

## Figures and Tables

**Table 1:** The top 10 differentially abundant genera and species in the Healthy vs. Diseased *P. polyphylla*.

Group	Genus	Mean (R. Healthy/S. Healthy)	Mean (R. Disease/S. Healthy)	*p*-Value
	*Lysinibacillus*	8.77 × 10^−05^	3.11 × 10^−05^	0.01
	unidentified_*Hydrogenedentes*	7.3 × 10^−05^	8.49 × 10^−06^	0.01
	unidentified_*Elusimicrobiota*	9.91 × 10^−05^	3.96 × 10^−05^	0.02
	*Edaphobaculum*	0.0005	6.79 × 10^−05^	0.02
R. Healthy vs. R. Disease	*Anaeromyxobacter*	0.0003	5.09 × 10^−05^	0.02
	unidentified_*Alphaproteobacteria*	0.0005	9.06 × 10^−05^	0.03
	*Halomonas*	0.0002	6.51 × 10^−05^	0.03
	unidentified_*Tepidisphaerales*	9.34 × 10^−05^	3.3 × 10^−05^	0.03
	unidentified_*Rhizobiales*	0.0028	0.0004	0.03
	*Muricauda*	0.0003	2.55 × 10^−05^	0.03
	unidentified_*Rhodospirillaceae*	0.0005	3.40 × 10^−05^	4.41 × 10^−08^
	*Pirellula*	0.0017	0.0003	5.80 × 10^−07^
	unidentified_*Chloroflexi*	0.0022	0.0002	7.73 × 10^−07^
	unidentified_*Alphaproteobacteria*	0.0021	9.06 × 10^−05^	1.26 × 10^−06^
	*Ferruginibacter*	0.0021	0.0001	1.41 × 10^−06^
S. Healthy vs. S. Disease	*Luteitalea*	0.0007	0.0001	2.41 × 10^−06^
	*Pajaroellobacter*	0.0042	0.0004	3.06 × 10^−06^
	*Edaphobaculum*	0.0025	6.79 × 10^−06^	4.86 × 10^−06^
	*Sandaracinus*	0.0001	1.13 × 10^−06^	5.36 × 10^−06^
	unidentified_*Micropepsaceae*	0.0015	0.0002	7.44 × 10^−06^
	*Lysinibacillus*	0.0015	5.09 × 10^−06^	1.16 × 10^−05^

**Table 2:** Identification of the top 20 differentially expressed genes in the Healthy vs. Diseased *P. polyphylla*.

Gene ID	Log2 FC	*p*-Value	H-2_fpkm	Regulation
Cluster-132319.1	5.839804085	9 × 10^−86^	2.84	up
Cluster-127001.21	−5.115231299	3.2 × 10^−75^	14,976.77	down
Cluster-122338.0	−5.240803144	2.8 × 10^−56^	24.91	down
Cluster-89170.0	−6.99819639	1 × 10^−50^	49.48	down
Cluster-126878.7	5.03189586	1.4 × 10^−41^	13.45	up
Cluster-25410.8	6.269167442	2.5 × 10^−41^	11.15	up
Cluster-127001.10	−2.899061791	7.3 × 10^−39^	13,951.98	down
Cluster-143583.18	8.710087805	8.2 × 10^−34^	6.47	up
Cluster-132771.4	4.376939557	3.3 × 10^−33^	4.21	up
Cluster-24639.4	−5.618000462	1.4 × 10^−31^	66.66	down
Cluster-124207.3	−2.490346547	8.4 × 10^−31^	139.07	down
Cluster-98045.0	15.95590433	7.3 × 10^−30^	0	up
Cluster-132856.60	−12.95953203	8.7 × 10^−29^	84.91	down
Cluster-115733.0	−8.138256466	1.9 × 10^−28^	205.57	down
Cluster-132856.91	7.690022495	5.8 × 10^−28^	7.3	up
Cluster-95921.0	3.168091923	7.6 × 10^−28^	14.28	up
Cluster-5756.5	−6.957003683	1.4 × 10^−27^	443.32	down
Cluster-45426.3	−15.9143436	2.9 × 10^−27^	448.77	down
Cluster-119914.0	−6.225555532	6.8 × 10^−27^	194.03	down
Cluster-104782.1	3.916116249	6.8 × 10^−27^	3.37	up

**Table 3:** Identification of the top 20 differentially abundant metabolites in the Healthy vs. Diseased *P. polyphylla*.

Compounds	VIP	*p*-Value	Fold_Change	Log_2_ FC	Regulation
Cyclo(Ser-Pro)	1.48	8.52 × 10^−08^	1.09 × 10^+04^	3.58 × 10^+00^	up
L-Aspartic acid-O-diglucoside	1.36	1.84 × 10^−07^	9.55 × 10^+03^	6.54 × 10^+00^	up
1-Eicosanol	1.49	7.12 × 10^−07^	8.66 × 10^+03^	3.41 × 10^+05^	up
Guanidinoacetate	1.42	6.62 × 10^−06^	8.37 × 10^+03^	3.51 × 10^+00^	up
Betaine	1.49	1.23 × 10^−05^	6.34 × 10^+03^	2.16 × 10^+06^	up
Hypoxanthine	1.39	1.62 × 10^−05^	4.59 × 10^+03^	2.58 × 10^+00^	up
Ricinoleic acid	1.49	2.13 × 10^−05^	3.96 × 10^+03^	2.87 × 10^−06^	down
1-O-(3,4-Dihydroxy-5-methoxy-benzoyl)-glucoside	1.41	2.59 × 10^−05^	3.36 × 10^+03^	3.07 × 10^+00^	up
5-Hydroxy-L-tryptophan	1.26	2.76 × 10^−05^	3.33 × 10^+03^	2.67 × 10^+00^	up
Naringenin (5,7,4′-Trihydroxyflavanone)	1.38	3.80 × 10^−05^	3.29 × 10^+03^	4.51 × 10^+00^	up
Naringenin-7-O-glucoside (Prunin)	1.15	7.78 × 10^−05^	2.35 × 10^−04^	2.71 × 10^+01^	up
Planteose	1.43	9.54 × 10^−05^	2.32 × 10^−04^	3.03 × 10^+00^	up
Ebeinone	1.32	1.13 × 10^−04^	1.88 × 10^−04^	3.89 × 10^+00^	up
L-Phenylalanine	1.38	1.30 × 10^−04^	1.51 × 10^−04^	2.59 × 10^+00^	up
6-O-Galloyl-β-D-glucose *	1.40	1.32 × 10^−04^	1.47 × 10^−04^	7.02 × 10^+00^	up
8-C-glucosyl-(R)-aloesol	1.19	1.39 × 10^−04^	1.06 × 10^−04^	4.91 × 10^+00^	up
Azetidine-2-carboxylic acid	1.30	1.49 × 10^−04^	1.00 × 10^−04^	3.22 × 10^+00^	up
γ-Linolenic Acid	1.49	1.51 × 10^−04^	5.96 × 10^−05^	4.22 × 10^+04^	up
Aminomalonic acid	1.37	1.51 × 10^−04^	3.12 × 10^−05^	4.33 × 10^−01^	down
4-Hydroxybenzaldehyde	1.38	1.61 × 10^−04^	3.19 × 10^−06^	4.50 × 10^+00^	up

Note: * represented the metabolite has isomerides.

## Data Availability

Data are contained within the article and Supplementary Materials.

## Supplementary Materials

The following supporting information can be downloaded at: https://www.mdpi.com/article/10.3390/genes15010021/s1, Figure S1: Total ion current (TIC) chromatograms and multiple reaction monitoring (MRM) metabolite detection multi-peak diagram (XIC of multi-substance extraction). (A. TIC, B. MRM.)
