# Supplementary material for: Multiomics Reveals the Key Microorganisms and Metabolites in the Resistance to Root Rot Disease of Paris polyphylla
Source: Genes (Basel). 2023 Dec 22;15(1):21. doi: 10.3390/genes15010021 (PMC10815090; doi:10.3390/genes15010021)
Supplement: Supplementary file 1 [file genes-2719565-supplementary.pdf › genes-2719565-supplementary.pdf]

A

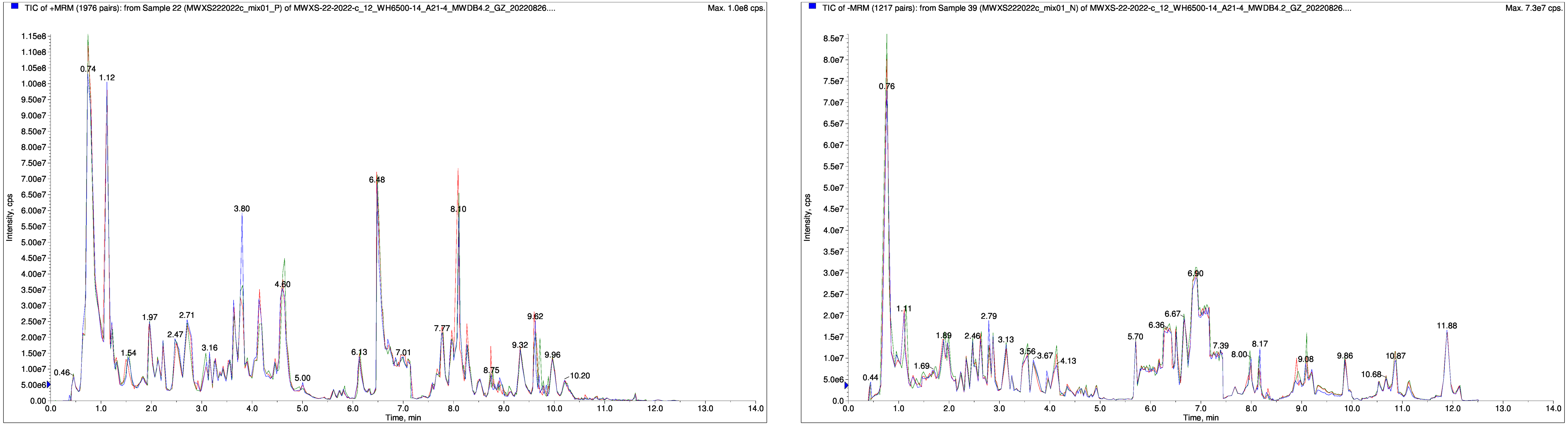
TIC of +MRM (1976 pairs): from Sample 22 (MWXS222022c_mix01_P) of MWXS-22-2022-c_12_WH6500-14_A21-4_MWDB4.2_GZ_20220826....
Max. 1.0e8 cps.
Intensity, cps
Time, min
0.46
0.74
1.12
1.54
1.97
2.47
2.71
3.16
3.80
4.60
5.00
6.13
6.48
7.01
7.77
8.10
8.75
9.32
9.62
9.96
10.20
TIC of -MRM (1217 pairs): from Sample 39 (MWXS222022c_mix01_N) of MWXS-22-2022-c_12_WH6500-14_A21-4_MWDB4.2_GZ_20220826....
Max. 7.3e7 cps.
Intensity, cps
Time, min
0.44
0.76
1.11
1.69
1.89
2.46
2.79
3.13
3.56
3.67
4.13
5.70
6.36
6.67
6.90
7.39
8.00
8.17
9.08
9.86
10.68
10.87
11.88

B

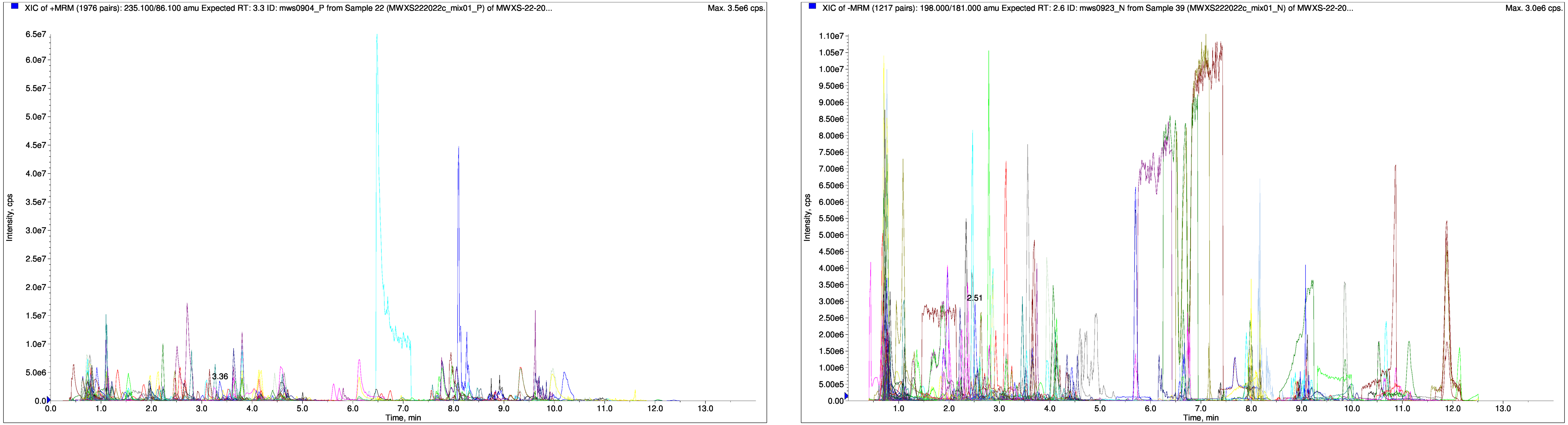
XIC of +MRM (1976 pairs): 235.100/86.100 amu Expected RT: 3.3 ID: mws0904_P from Sample 22 (MWXS222022c_mix01_P) of MWXS-22-20...
Max. 3.5e6 cps.
Intensity, cps
Time, min
3.36
XIC of -MRM (1217 pairs): 198.000/181.000 amu Expected RT: 2.6 ID: mws0923_N from Sample 39 (MWXS222022c_mix01_N) of MWXS-22-20...
Max. 3.0e6 cps.
Intensity, cps
Time, min
2.51
